# Supplementary material for: Deep visual proteomics reveals DNA replication stress as a hallmark of signet ring cell carcinoma
Source: NPJ Precis Oncol. 2025 Feb 5;9:37. doi: 10.1038/s41698-025-00819-7 (PMC11799539; doi:10.1038/s41698-025-00819-7)
Supplement: Supplementary file 1 — Supplementary Figure 1 [file 41698_2025_819_MOESM1_ESM.pdf]

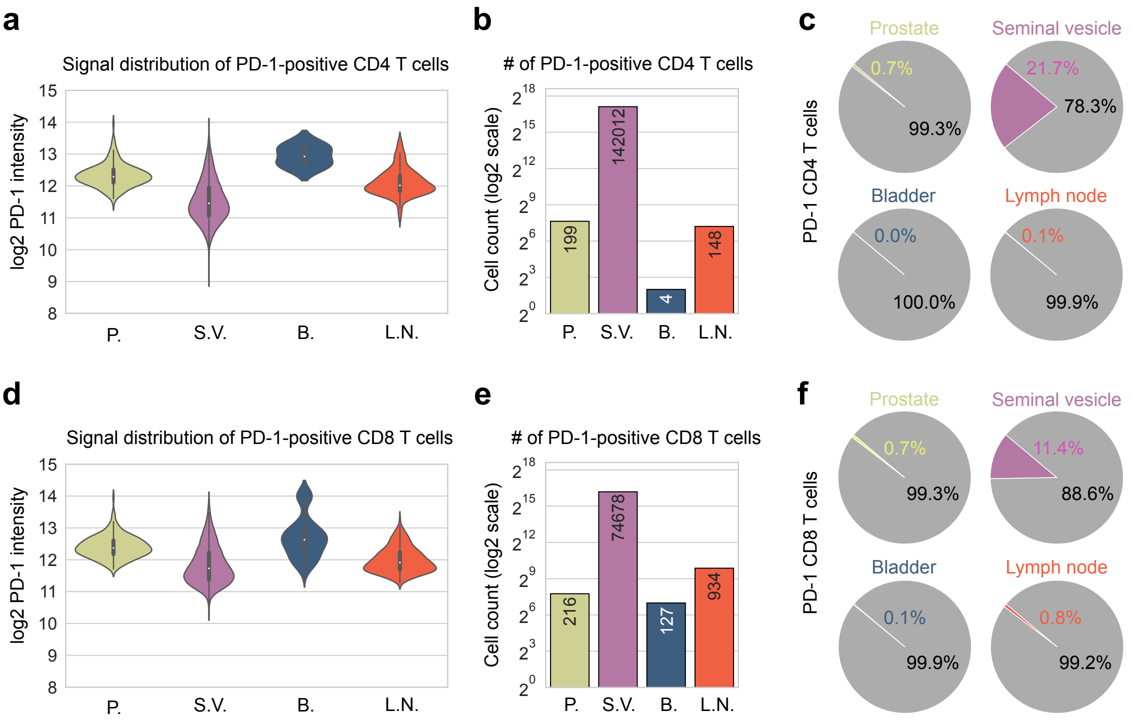

g

PD-1 in T cell	Comparison	U statistic	P-Value	Corrected P-Value (Benjamini-Hochberg)	Significant
CD8 T cells	Prostate vs Seminal vesicle	12833247.0	4.869 e-51	2.921 e-50	TRUE
CD8 T cells	Prostate vs Bladder	9927.0	1.936 e-05	2.581 e-05	TRUE
CD8 T cells	Prostate vs Lymph node	159049.0	6.282 e-40	1.885 e-39	TRUE
CD8 T cells	Seminal vesicle vs Bladder	1516765.5	3.716 e-40	1.487 e-39	TRUE
CD8 T cells	Seminal vesicle vs Lymph node	26935363.5	4.761 e-33	9.522 e-33	TRUE
CD8 T cells	Bladder vs Lymph node	98891.0	2.539 e-34	6.094 e-34	TRUE
CD4 T cells	Prostate vs Seminal vesicle	24033379.0	1.202 e-65	1.442 e-64	TRUE
CD4 T cells	Prostate vs Bladder	63.0	0.001	0.001	TRUE
CD4 T cells	Prostate vs Lymph node	19918.0	1.938 e-08	2.908 e-08	TRUE
CD4 T cells	Seminal vesicle vs Bladder	20900.0	0.002	0.001	TRUE
CD4 T cells	Seminal vesicle vs Lymph node	4613898.0	3.312 e-32	5.678 e-32	TRUE
CD4 T cells	Bladder vs Lymph node	552.0	0.001	0.001	TRUE

Supplementary Fig. 1 Quantification of PD-1 signal on CD4- and CD8-positive T cells across prostate, seminal vesicle, bladder, and lymph node tissues.
a Distribution of PD-1 signal in CD4-positive T cells. **b** Number of PD-1-positive CD4 T cells. **c** Percentage of PD-1-positive CD4-positive T cells within each tissue, normalized to all segmented cells (shown in gray). **d** Distribution of PD-1 signal in CD8-positive T cells. **e** Number of PD-1-positive CD8-positive T cells. **f** Percentage of PD-1-positive CD8-positive T cells within each tissue, normalized to all segmented cells (shown in gray). **g** Statistical comparison of PD-1 signal between CD4- and CD8-positive T cells across tissues, performed using pairwise Mann-Whitney tests with an FDR threshold of 0.01 and Benjamini-Hochberg multiple hypothesis correction.
